# Correction: Incidence of dengue illness in Mexican people aged 6 months to 50 years old: A prospective cohort study conducted in Jalisco

**DOI:** 10.1371/journal.pone.0252636

**Published:** 2021-05-27

**Authors:** The PLOS ONE Staff

## Notice of republication

An incorrect version of S1 and S2 Appendices were published in error. The publisher apologizes for this error. This article was republished on May 14, 2021 to correct for this error. Please download this article again to view the correct version.
