# Supplementary material for: Incidence of dengue illness in Mexican people aged 6 months to 50 years old: A prospective cohort study conducted in Jalisco
Source: PLoS One. 2021 May 5;16(5):e0250253. doi: 10.1371/journal.pone.0250253 (PMC8099064; doi:10.1371/journal.pone.0250253)
Supplement: S1 Appendix — (PDF) [file pone.0250253.s001.pdf]

Protocolo: 200318 (EPI-DENGUE-007 BOD)

Nº Familia: Fecha:

Nº de Participantes:

Características de la Vivienda

1. Número de personas de viven en la casa

   Adultos:
   Niños:

2. Tipo de vivienda
   - ☐ Casa
   - ☐ Departamento

3. Número de cuartos:

4. Número de dormitorios:

5. Recibe agua del sistema público

   Días por semana:
   - ☐ < 2
   - ☐ 2-5
   - ☐ 6-7

   Horas por día:
   - ☐ < 3
   - ☐ 3-12
   - ☐ 13-24

6. Otras fuentes de suministro de agua
   - ☐ Deposito de agua/cisterna/tinaco
   - ☐ Pozo

7. Locación de la casa
   - ☐ Área urbana
   - ☐ Área rural

8. Material del techo de la casa
- ☐ Galvanizado / aluminio / estaño
- ☐ Paja / paja
- ☐ Concreto / Cemento
- ☐ Tablones de madera
- ☐ Improvisado / cartón
- ☐ Azulejos de cerámica
- ☐ Otros, especificar:

9. Material de las paredes externas de la vivienda
- ☐ bloques de cemento / cemento /concreto
- ☐ hormigón de madera
- ☐ tablas de madera
- ☐ yute palillo / de bambú / caña / troncos
- ☐ hierro galvanizado / aluminio / estaño
- ☐ concreto / cemento
- ☐ Otro, especificar:

10. Material del piso
- ☐ Cemento
- ☐ Azulejos de cerámica
- ☐ Tablones de madera
- ☐ Palma / bambú
- ☐ Tierra / arena
- ☐ Vinil o asfalto tiras
- ☐ Parquet o madera pulida
- ☐ Otro, especificar:
