# Supplementary material for: Incidence of dengue illness in Mexican people aged 6 months to 50 years old: A prospective cohort study conducted in Jalisco
Source: PLoS One. 2021 May 5;16(5):e0250253. doi: 10.1371/journal.pone.0250253 (PMC8099064; doi:10.1371/journal.pone.0250253)
Supplement: S2 Appendix — Cases definitions and sample size calculation. (DOCX) [file pone.0250253.s002.docx]

# Supplementary Appendix 2

## Supplementary Methods – case definitions

### Suspected Dengue Case

- Body temperature ≥38.0°C lasting 1.5 to 7 days within the past eight days
- Other dengue-associated signs in infants and older children included (but were not limited to): upper respiratory tract and gastrointestinal symptoms as well as febrile convulsion.
- Other dengue-associated signs in adolescents and adults included (but were not limited to): fatigue, headache, pain behind the eyes, abdominal pain, nausea, vomiting, muscle ache, joint ache, diffuse rash on the trunk, and pruritis.

### Warning signs

- *Dengue cases with warning signs* had at least one of the following symptoms: abdominal pain or tenderness, persistent vomiting, clinical fluid accumulation, mucosal bleeding, liver enlargement, increase in hematocrit concurrent with a rapid decrease in platelet count, lethargy, restlessness.

### Severe symptoms

- *Severe dengue* had at least one of the following symptoms: severe plasma leakage leading to shock, fluid accumulation with respiratory distress, severe bleeding, severe organ involvement, failure of heart and other organs.

## Supplementary Methods – sample size calculation

The target population was estimated at 1,750 overall participants (approximately 300 to 500 per site). A dropout rate of 5% per year was estimated, which would lead to approximately 1,662 participants completing the first year of follow-up and approximately 1,579 completing two years of follow-up, accumulating 3,327 patient-years.

The incidence of dengue was likely to vary by site and by age group. The study population should thus include between 30% and 50% of adults, but the distribution was not further specified as it would make operational feasibility more complex.

Supposing that the study population was composed of 70% of children with an expected incidence of 8 RT-qPCR-confirmed dengue cases per 1,000 person-years and 30% of adults with an expected incidence of 5 RT-qPCR confirmed dengue cases per 1,000 person-years, the study would detect about 19 cases in children and 5 cases in adults (12 cases in Year one with a cohort of 1,750 subjects and 12 cases in Year 2 with a cohort of about 1,662 subjects). The overall incidence rate would be 7.1 per 1,000 person-years with an exact Poisson 95% confidence interval (CI) of [4.5; 10.6] and with a CI based on the normal approximation and accounting for the design effect of [3.3; 10.9].

The enrolment was done by household. Each household could be considered as a cluster and this induced a design effect to account for the between-cluster variability when estimating the CI of the incidence rates. The design effect measures the increase in the standard error of the incidence rate estimate due to the sampling design used and is given by D = 1 + (b – 1) rho, where rho is the intra-cluster correlation (a measure of the rate of homogeneity within clusters) and b is the average number of subjects sampled per household. Here, b was assumed to be 3. Although in theory ‘rho’ can have a value up to 1, in practice values higher than 0.4 are uncommon. A conservative estimate of 0.4 was used for this study. The design effect was then estimated to be 1.8.
