# Supplementary material for: Incidence of dengue illness in Mexican people aged 6 months to 50 years old: A prospective cohort study conducted in Jalisco
Source: PLoS One. 2021 May 5;16(5):e0250253. doi: 10.1371/journal.pone.0250253 (PMC8099064; doi:10.1371/journal.pone.0250253)
Supplement: Attachment — Submitted filename: PONE-D-20-24564R1_Comments due 15 Jan 2021.docx [file pone.0250253.s004.docx]

PONE-D-20-24564R1_	Accepted January 05 2021

Due January 15 2021

Submitted January 13 2021

Incidence of dengue illness in Mexican people aged 6 months to 50 years old: results from a prospective, cohort study conducted in the Jalisco state

Comments:

**Methods**

LINES 135-136

However, due to early study termination, only two sites in the Philippines and Mexico participated in the study.

LINES 146-47

However, due to early study termination, only two sites in the Philippines and Mexico participated in the study.

Mention this sentence just once in the paper.

If the Philippines participated, where are the data and the analyses? Please clarify

Expound briefly on “early study termination”

Page 16 Top row

Conjunctivis (spelling)

Page 12 Table 1

Age groups: The authors listed 5 levels, 1-4 and 5-8 years could be combined as they did not differ much in values and that the range would be closer to 9-17 and 18-50 years

LINE 256 household ranged from one to seven (median: 4).

The authors reported IQR in Table 1 but not in LINE 256

LINES 247-248 incidence proportions. All statistical analyses were performed using the statistical analysis systems version 9.4. Cite the source of the system used

LINE 233 Statistical analysis was performed on all evaluable eligible participants What is evaluable?
