# Supplementary material for: Incidence of dengue illness in Mexican people aged 6 months to 50 years old: A prospective cohort study conducted in Jalisco
Source: PLoS One. 2021 May 5;16(5):e0250253. doi: 10.1371/journal.pone.0250253 (PMC8099064; doi:10.1371/journal.pone.0250253)
Supplement: Attachment — Submitted filename: DeAntonio et al - response letter.docx [file pone.0250253.s005.docx]

March 30, 2021

Dear Editor,

We would like to thank you for the consideration of our revised manuscript entitled “*Burden of dengue illness in Mexican people aged 6 months to 50 years old: results from a prospective, cohort study conducted in the Zapopan community”* (PONE-D-20-24564R1).

We have addressed all additional comments from the reviewers below and revised the manuscript using track-changes. We hope that the revised manuscript will fully meet PLOS ONE’s publication criteria.

We look forward to future correspondence regarding our submission and are more than happy to provide further information on any questions or comments you may have.

With kind regards,

*Melanie de Boer*

GSK
Email	[melanie.x.de-boer@gsk.com](mailto:melanie.x.de-boer@gsk.com)

# Comments:

## Reviewer #2

-Throughout the paper, the author should write either “the state of Jalisco” or simply “Jalisco” instead of “Jalisco state”

**Reply:** We made the correction throughout the text

-Suggested title: “Incidence of dengue illness in Mexican people aged 6 months to 50 years old: a prospective cohort study conducted in Jalisco”

**Reply:** We have modified the title as suggested.

-Line 41: no space between the number 19.4 and %

**Reply:** The space between numerals and the % has been removed (line 38 of the revised version with tracked changes).

-Line 46: “cases were tested negative by these assay”

**Reply:** We have revised the sentence as suggested (line 43 of the revised version with tracked changes).

-Line 49: delete the word “conduct”

**Reply:** Deleted (line 46 of the revised version with tracked changes)

-Line 70: “Worldwide, the global incidence of dengue”

**Reply:** We have inserted “global” in the sentence (line 67 of the revised version with tracked changes).

-Line 85-88: these three sentences are all simple sentences and should be reworded and/or combined together

**Reply:** We have revised the sentences to improve the flow (lines 81-87 of the revised version with tracked changes).

-Line 112-113: the phrase “using serotype-specific polymerase chain reaction (PCR) and anti-dengue immunoglobulin G (DENV IgG) assays” is not relevant to the previous clause in the sentence. The author may consider deleting that phrase.

**Reply:** We have deleted that part of the sentence (lines 111-112 of the revised version with tracked changes).

-Line 124: replace the word “vaccinees” by “vaccinated people” or “vaccinated individuals”

**Reply:** “Vaccinees” has been changed for “vaccinated individuals” (line 120 of the revised version with tracked changes).

-Line 146: 14th, 2016 and September 14th, 2018.

**Reply:** Corrected (lines 141-142 of the revised version with tracked changes).

-Line 177: replace the word “take” by “measure”

**Reply:** Replaced as suggested (line 168 of the revised version with tracked changes).

-Line 219: the author may consider changing the phrase “As an alternative to the combined test was not available” to “Due to the unavailability of the combined test”

**Reply:** Revised as suggested (line 208 of the revised version with tracked changes).

-Line 270: no comma between “(69.4%)” and “and Guadalajara”

**Reply:** Corrected (line 257 of the revised version with tracked changes).

-Line 271: should have a comma between “Tlajomulco” and “and Chapala”

**Reply:** Corrected (line 38 of the revised version with tracked changes).

-Line 272: should not begin a sentence by a number

**Reply:** Corrected (line 259 of the revised version with tracked changes).

-Line 355: “dengue in Jalisco, in an area where”

**Reply:** We have removed “in” before “an area where” (line 342 of the revised version with tracked changes).

-Line 364: the author can consider adding a subheader called “Limitations”

**Reply:** We have added the subheading for limitations (line 350 of the revised version with tracked changes).

-Line 380: change the word “successful” to “effective”

**Reply:** Revised as suggested (line 366 of the revised version with tracked changes).

## Reviewer #4

**Methods**

LINES 135-136

However, due to early study termination, only two sites in the Philippines and Mexico participated in the study.

LINES 146-47

However, due to early study termination, only two sites in the Philippines and Mexico participated in the study.

Mention this sentence just once in the paper.

**Reply:** We have deleted the sentence from lines 146-47 (lines 149-151 of the revised version with tracked changes)

If the Philippines participated, where are the data and the analyses? Please clarify

Expound briefly on “early study termination”

**Reply:** As the global, pre-defined analysis could not be performed, data from the Philippines and Mexico were analyzed separately. Data and results for the Philippines are described in another paper currently under consideration for publication in *Asian Biomedicine*.

In December 2017, GSK notified its longstanding collaboration partners, Institution Oswaldo Cruz (Fiocruz) and the Walter Reed Army Institute of Research and the US Army Medical Materiel Development Agency (WRAIR/USAMMDA), of the intention of GSK’s Vaccines Investment Board (VIB) to deprioritize the development of the dengue purified inactivated vaccine (DPIV) candidate. This decision was made due to the scientific challenges and development risks associated with the candidate vaccine. This decision entailed the early termination of the associated observational studies sponsored by GSK.

Page 16 Top row

Conjunctivis (spelling)

**Reply:** Corrected to “conjunctivitis”, thank you!

Page 12 Table 1

Age groups: The authors listed 5 levels, 1-4 and 5-8 years could be combined as they did not differ much in values and that the range would be closer to 9-17 and 18-50 years

**Reply:** We acknowledge that 1-4 and 5-8 age groups do not differ much in values and could have been combined. However, the stratification was made as per protocol and merging both group would require an additional analysis that would take time while having little impact on our observations and the overall message of the paper.

LINE 256 household ranged from one to seven (median: 4).

The authors reported IQR in Table 1 but not in LINE 256

**Reply:** IQR is provided is Table 1 for the median age at enrolment. We have nevertheless added the IQR in text for the number of participants by household (lines 261-262 of the revised version with tracked changes).

LINES 247-248 incidence proportions. All statistical analyses were performed using the statistical analysis systems version 9.4. Cite the source of the system used

**Reply:** We have added the software, SAS, in brackets in the sentence (line 253 of the revised version with tracked changes).

LINE 233 Statistical analysis was performed on all evaluable eligible participants

What is evaluable?

**Reply:** We have removed evaluable and inserted “with complete data” instead. We hope that this revision clarifies the meaning of the sentence (lines 238-239 of the revised version with tracked changes).
